# Comparison of the Cortical Activity Index and Bispectral Index During General Anesthesia: A Prospective Observational Study

**DOI:** 10.3390/medicina62081545

**Published:** 2026-08-12

**Authors:** Tae-Yun Sung, Seokjin Lee, Choon-Kyu Cho, Ji-Yoon Jung, Hojin Oh, Seokhee Hwang, Woojin Kwon

**Affiliations:** 1 Department of Anesthesiology and Pain Medicine, Konyang University Hospital, Konyang University College of Medicine, Daejeon 35365, Republic of Korea; 2 Myunggok Medical Research Institute, Konyang University Hospital, Konyang University College of Medicine, Daejeon 35365, Republic of Korea

**Keywords:** bispectral index, cortical activity index, electroencephalography, depth of anesthesia, general anesthesia

## Abstract

*Background and Objectives*: No processed electroencephalographic index is a gold standard for anesthetic depth. We compared the Cortical Activity Index (CAI) with the bispectral index (BIS) as a pragmatic, widely studied clinical comparator, without assuming that BIS measures the true hypnotic state or that CAI is superior. *Materials and Methods*: This single-center prospective observational study enrolled 100 adult surgical patients; seven cases with acquisition-device data loss were excluded according to the final research-results report, leaving 93 participants for analysis. Concurrent CAI and BIS values were evaluated across 31 analyzable perioperative stages. The prespecified similarity criterion was a mean patient-level Pearson correlation coefficient of at least 0.85. *Results*: After protocol-specified last observation carried forward handling, 2876 paired CAI-BIS observations were available. The mean patient-level correlation was 0.778 (SD 0.200), with a median of 0.845 (IQR 0.716–0.913); therefore, the prespecified similarity criterion was not met. The pooled observation-level correlation was 0.750. Both indices showed broadly similar perioperative trajectories, but BIS values were generally higher, and intubation-period spikes occurred more often for BIS than CAI (73.1% vs. 19.4%). *Conclusions*: Comparison with BIS provided a clinically relevant benchmark for CAI behavior but did not establish the absolute accuracy or superiority of either index. CAI did not meet the prespecified similarity criterion, supporting further validation against independent EEG and clinical endpoints.

## 1. Introduction

Accurate assessment of the hypnotic component of anesthesia remains difficult. Clinical signs are indirect and can be altered by opioids, neuromuscular blockade, and autonomic drugs. Processed electroencephalographic (EEG) indices provide additional information about cerebral drug effects, but none constitutes a gold standard or directly measures consciousness. BIS (Medtronic, Boulder, CO, USA) is the most extensively studied and commonly used processed EEG index, with a conventional maintenance range of 40–60, yet its output also depends on proprietary algorithms and can be affected by anesthetic technique, electromyographic activity, electrical artifact, and patient factors [1,2,3,4,5].

CAI (BrainU Co., Ltd., Seoul, Republic of Korea) is a processed EEG index derived from brain-activity signal modeling and quantification of peak-signal component density, with higher values intended to reflect greater cortical activity [6]. Clinical evidence remains limited. Huh et al. reported a CAI-BIS correlation of 0.825 in 32 patients undergoing laparoscopic cholecystectomy [6]. That study did not establish a general-anesthesia CAI target, demonstrate improved outcomes with CAI-guided care, or determine whether CAI and BIS respond similarly across heterogeneous procedures and stimulation-intensive events.

BIS was therefore selected as a pragmatic clinical comparator, not as a gold standard. Simultaneous comparison with the monitor used to guide anesthetic titration can characterize CAI’s convergent perioperative behavior, systematic numeric offset, and event-specific divergence. Such a comparison cannot determine which index is closer to the true hypnotic state, and correlation cannot establish agreement, interchangeability, or superiority. Independent validation against raw EEG features and clinical outcomes remains necessary.

The primary aim of this prospective observational study was to quantify patient-level CAI-BIS correlation across predefined perioperative stages and determine whether the prespecified mean correlation criterion of r ≥ 0.85 was met. Secondary analyses evaluated pooled observation-level correlation, absolute agreement, stage-dependent trajectories, anesthetic-technique subgroups, and divergence during intubation and low-BIS episodes. The study was not designed to demonstrate CAI superiority or to validate BIS as a gold standard.

## 2. Materials and Methods

### 2.1. Study Design and Participants

This was a single-center, prospective observational repeated-measures study conducted at Konyang University Hospital. Participants were enrolled consecutively from 31 October 2021 through 30 June 2022. The study was approved by the Konyang University Hospital Institutional Review Board (IRB No. 2021-06-007; initial approval date, 23 July 2021), and the IRB-approved study period was 23 July 2021 through 22 July 2022. Written informed consent was obtained from all participants. This report was prepared in accordance with the Strengthening the Reporting of Observational Studies in Epidemiology (STROBE) guideline for cohort studies.

Eligible participants were male or female patients aged 20–75 years who were scheduled to undergo surgery under general anesthesia and had an American Society of Anesthesiologists physical status of I–III. Patients who did not provide consent or had an ASA physical status of IV or higher were excluded. Eligible patients were approached consecutively during the recruitment period. The protocol specified a target of 100 participants from the outset, based on methodological guidance indicating that approximately 25–50 paired datasets were needed for correlation analysis rather than on a formal power calculation. The final research-results report documented 100 screened and registered participants, no screening failures, seven exclusions caused by acquisition-device data loss, and 93 participants in the final analysis.

### 2.2. Study Discontinuation and Exclusion Criteria

According to the protocol, study participation could be discontinued if the participant withdrew consent, if an intraoperative change in clinical condition or adverse event made continued participation difficult, if a serious protocol violation occurred, or if either the CAI or BIS sensor detached three or more times during surgery. Participants could be excluded from analysis if EEG analysis errors occurred in at least 20% of the analyzable data. For participants with EEG analysis errors, the protocol also recorded whether the awake baseline sublingual temperature was ≤36 degrees C because lower body temperature can alter EEG activity; this threshold was a protocol-defined screening variable rather than a validated CAI- or BIS-specific exclusion threshold.

For discontinued or excluded participants, protocol-related data collected up to the time of discontinuation or exclusion were to be recorded and retained. At study closure, cases 24 and 39 had initially been categorized as dropouts because of intraoperative discontinuation and BIS-sensor difficulty, respectively. Subsequent source-data review found their available records usable, and both were retained in the final analysis. The final research-results report identified cases 15–20 and 58 as unavailable because of acquisition-device data loss; these seven cases were excluded, leaving 93 participants. The protocol specified last observation carried forward (LOCF) handling for intermittent missing efficacy values within included participants; values without a preceding observation were left missing.

### 2.3. Monitoring and Anesthetic Management

After arrival in the operating room, standard safety monitoring, including noninvasive blood pressure, pulse oximetry, and electrocardiography, was applied. The BIS-Vista sensor was placed on the left side of the forehead, and the CAI sensor was placed on the right side of the forehead, simultaneously and according to the respective manufacturers’ recommendations. The BIS-Vista monitor was connected and the EEG waveform and BIS value were confirmed before induction; a separate USB storage device was connected to the BIS-Vista monitor for EEG data storage. The CAI monitor was then connected to its sensor and amplifier, and the EEG waveform and CAI value were confirmed before induction.

General anesthesia was induced and maintained according to the anesthetic technique selected by the attending anesthesiologist on the basis of clinical judgment and surgical requirements; there was no randomization or prespecified allocation ratio. In the balanced anesthesia group, anesthesia was induced with propofol 2 mg/kg, sufentanil delivered by target-controlled infusion at an initial effect-site concentration of 0.3 ng/mL, and rocuronium 0.8 mg/kg. Anesthesia was maintained with sevoflurane and sufentanil, which were adjusted according to BIS values and clinical judgment. In the TIVA group, induction and maintenance were performed from the outset with target-controlled infusions of propofol and remifentanil, without a preceding manual propofol bolus. Initial effect-site targets were 3.0 mcg/mL for propofol using the Schnider model and 3.0 ng/mL for remifentanil using the Minto model, and were subsequently adjusted to maintain BIS values between 40 and 60. Rocuronium 0.8 mg/kg was administered for neuromuscular blockade. Ketamine, dexmedetomidine, midazolam, and magnesium were not administered.

Hemodynamic management was performed similarly in both anesthesia groups. Hypotension, defined as systolic blood pressure ≤ 100 mmHg or a decrease of at least 30% from the patient’s usual or baseline systolic blood pressure, was treated with phenylephrine initiated at 0.3 mcg/kg/min and increased as needed. Hypertension, defined as systolic blood pressure ≥ 150 mmHg or an increase of at least 30% from the patient’s usual or baseline systolic blood pressure, was treated with nicardipine 0.5 mg boluses. Anesthetic administration was adjusted using the BIS target range of 40–60 together with usual clinical judgment. CAI values were recorded concurrently but were not used to guide anesthetic titration. BrainU Co., Ltd., the manufacturer of the CAI monitor, provided the CAI monitor, CAI sensors, and related consumables as in-kind support and assisted with technical extraction and the compilation of monitor and CRF values into an Excel-format dataset. The investigators retained the source data. Seokjin Lee independently cross-checked the Excel dataset against the source data and found no material discrepancies. BrainU did not perform analytic data cleaning, determine exclusions, conduct statistical analyses, interpret the results, or participate in manuscript preparation. Analytic cleaning and statistical analysis were performed independently by the investigators.

### 2.4. Recording Time Points and Data Processing

The study protocol defined 36 planned perioperative recording checkpoints, spanning awake baseline, induction and neuromuscular blockade, intubation, surgery start, 5 min after incision, surgery end, emergence, recovery events, extubation, and departure from the operating room. The final master CRF contained 31 analyzable stage sheets because the five planned reversal-agent checkpoints were not present as separate sheets; subsequent recovery events were therefore renumbered as analytic stages 22–31.

Frontal EEG signal acquisition was intermittently degraded by electrical interference during electrocautery and operation of the forced-air warming device. Airway manipulation, coughing, facial movement, and sensor displacement could also degrade signal quality, particularly after extubation. All 31 recorded analytic stages in the master CRF were retained in the prespecified primary analysis. Independent adjudication of raw EEG, electromyographic activity, and device signal-quality indices was not performed, and the specific technical cause of each missing value could not be determined retrospectively. Non-numeric values, including values entered as X, were treated as missing before protocol-specified LOCF imputation of CAI and BIS efficacy values. The patient registration number field was removed from analytic outputs; analyses used only the study sequence number. BMI was calculated from height and weight when the CRF BMI field was blank.

### 2.5. Statistical Analysis

Continuous variables are summarized as mean (SD), and categorical variables as number (percentage). The primary endpoint was the patient-level Pearson correlation coefficient between CAI and BIS across CRF stages. The mean patient-level threshold of r ≥ 0.85 was specified a priori in the study protocol. This threshold was selected to require close parallel tracking: an r of 0.85 corresponds to approximately 72% shared variance (r squared = 0.7225), making it substantially more demanding than simply demonstrating a moderate or strong association. It remained a stringent operational similarity benchmark, not a validated clinical cutoff, and does not establish agreement or interchangeability. Patient-level correlations were summarized overall and by anesthetic technique. Pooled observation-level correlations and stage-order correlations were secondary descriptive analyses. Patient-level agreement was summarized using the mean BIS-CAI difference, mean absolute error (MAE), and root mean square error (RMSE). The TIVA subgroup analyses were exploratory and descriptive because technique was not randomized and the groups were markedly unbalanced. The intubation-spike analysis was post hoc; a 10-point increase was chosen as a readily interpretable change on the 0–100 index scale, not as a validated clinical threshold. Missing efficacy values were handled using protocol-specified LOCF within each participant in stage order. A complete-case sensitivity analysis repeated the main patient-level and pooled correlations after excluding every imputed observation. Because the primary criterion was not met, interpretation emphasizes descriptive concordance and divergence rather than confirmatory non-inferiority, equivalence, or technique-specific performance.

## 3. Results

### 3.1. Patient Flow and Analysis Set

One hundred participants were screened and registered, with no screening failures. Seven cases (15–20 and 58) were excluded from analysis because acquisition-device errors caused data loss, leaving 93 participants (75 who received balanced anesthesia and 18 who received TIVA). The analytic dataset therefore contained 2883 potential stage observations across 31 stages. Protocol-specified LOCF imputation replaced 8 missing CAI values and 15 missing BIS values. After LOCF handling, 2876 complete paired CAI-BIS observations were available; 7 initial BIS observations remained missing because no preceding value was available for carry-forward imputation. Participant flow is shown in the submitted flow diagram.

Seventy-five patients received balanced anesthesia and 18 received TIVA. Baseline and perioperative characteristics are summarized in Table 1.

### 3.2. Primary Endpoint and CAI-BIS Correlation

Across the 31 analyzable CRF stages, the mean patient-level Pearson correlation between CAI and BIS was 0.778 (SD 0.200), with a median of 0.845 (IQR 0.716–0.913). This result did not meet the prespecified primary success criterion of mean patient-level r ≥ 0.85. Correlation describes parallel variation but does not establish numeric agreement or interchangeability.

Forty-six patients (49.5%) had a patient-level correlation coefficient of at least 0.85. The mean patient-level correlation was 0.794 in balanced anesthesia and 0.715 in TIVA; because only 18 patients received TIVA and technique was not randomized, these subgroup findings are descriptive and do not support a reliable comparison of monitor performance between techniques. The pooled observation-level CAI-BIS correlation across all complete paired observations after LOCF was 0.750 (95% CI 0.734–0.766).

**Figure 1:**
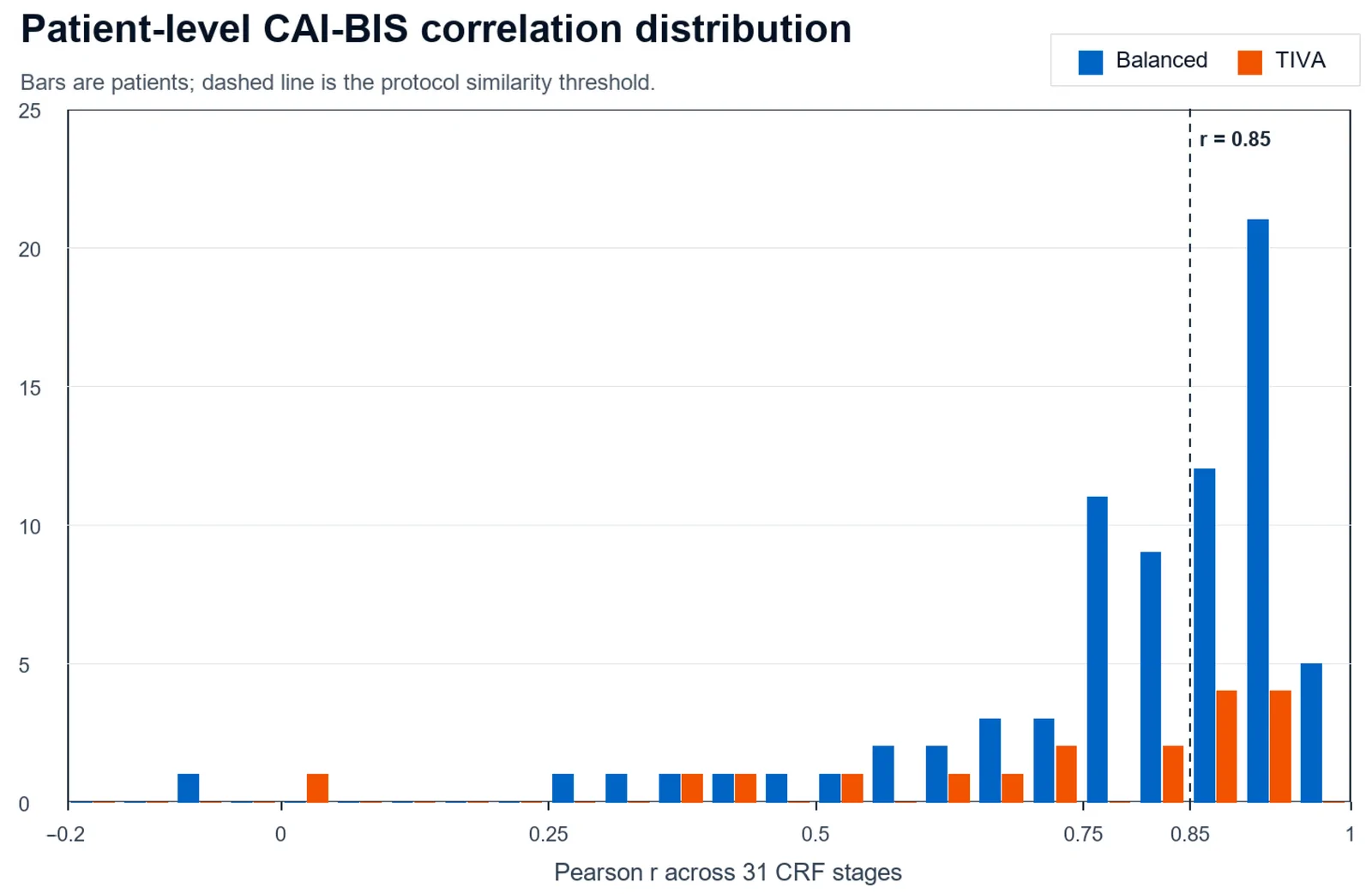
Distribution of patient-level CAI-BIS Pearson correlation coefficients across 31 analyzable stages. The dashed line indicates the prespecified similarity threshold of r = 0.85.

In the complete-case sensitivity analysis, 2861 originally observed pairs were available. The mean patient-level correlation was 0.779 (SD 0.199), with a median of 0.843 (IQR 0.739–0.913), and the pooled observation-level correlation was 0.751 (95% CI 0.735–0.767). These results were essentially unchanged from the protocol-specified LOCF analysis. The primary, pooled, and sensitivity analyses are summarized in Table 2 and Figure 1.

### 3.3. Agreement and Index Difference

Across patients, BIS values were generally higher than CAI values. The mean patient-level BIS-CAI difference was 7.6 score points (SD 6.5), with a mean absolute error of 12.4 (SD 4.6) and RMSE of 15.3 (SD 5.5). This offset means that correlation should not be interpreted as numeric interchangeability: the two indices moved together over time, but their absolute values were not identical.

### 3.4. Stage-Dependent Trajectories

To distinguish physiologically different periods, stages 1–13 were summarized as induction/intubation, stages 14–16 as intraoperative maintenance, and stages 17–31 as emergence/recovery. Across paired observations, mean CAI/BIS values were 52.2/58.2 during induction/intubation, 42.5/46.3 during intraoperative maintenance, and 64.5/74.1 during emergence/recovery; the corresponding pooled CAI-BIS correlations were 0.739, 0.415, and 0.691. These phase-level correlations are descriptive and do not establish agreement.

**Figure 2:**
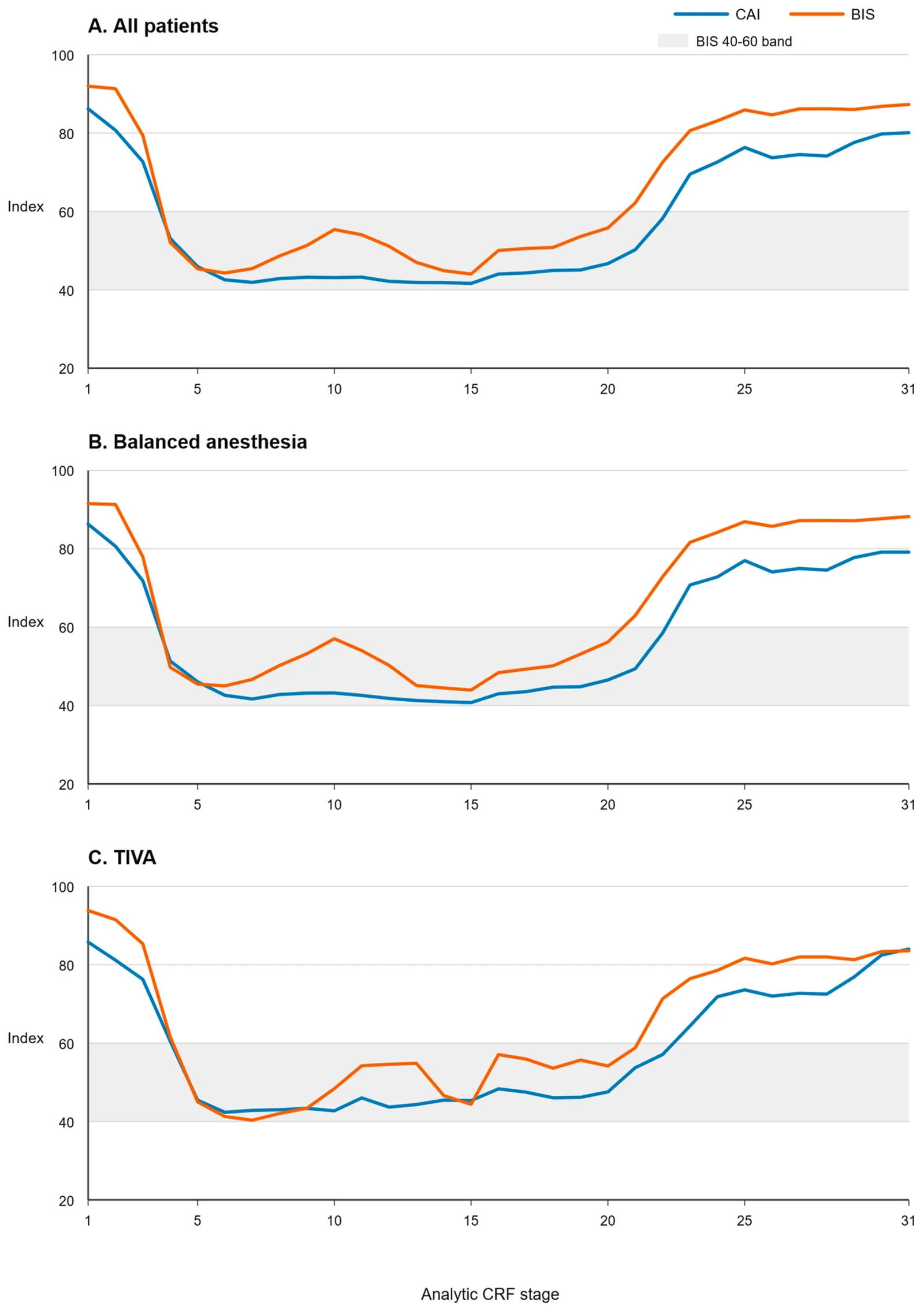
Mean CAI and BIS trajectories across 31 analyzable perioperative stages. The shaded band indicates the conventional BIS maintenance range of 40–60. (**A**) All patients; (**B**) balanced anesthesia; (**C**) TIVA.

With advancing stage order, both indices decreased during induction/intubation (CAI r = −0.658; BIS r = −0.527), changed little across the three available intraoperative maintenance checkpoints (CAI r = 0.133; BIS r = 0.235), and increased during emergence/recovery (CAI r = 0.646; BIS r = 0.803). The maintenance dataset contained only surgery start, 5 min after incision, and surgery end; no scheduled mid-surgery measurement was available. The complete stage-specific trajectories are shown in Figure 2.

### 3.5. Intubation-Period Divergence

During the intubation period, a post-intubation increase of at least 10 index points occurred more often for BIS than for CAI. Overall, BIS showed a spike in 68/93 (73.1%) patients, whereas CAI showed a spike in 18/93 (19.4%) patients. This was a post hoc descriptive analysis using a nonvalidated threshold. Without raw EEG, EMG, or signal-quality adjudication, the observed differences cannot be classified as physiological responses or artifacts.

### 3.6. BIS Values Below 35

**Figure 3:**
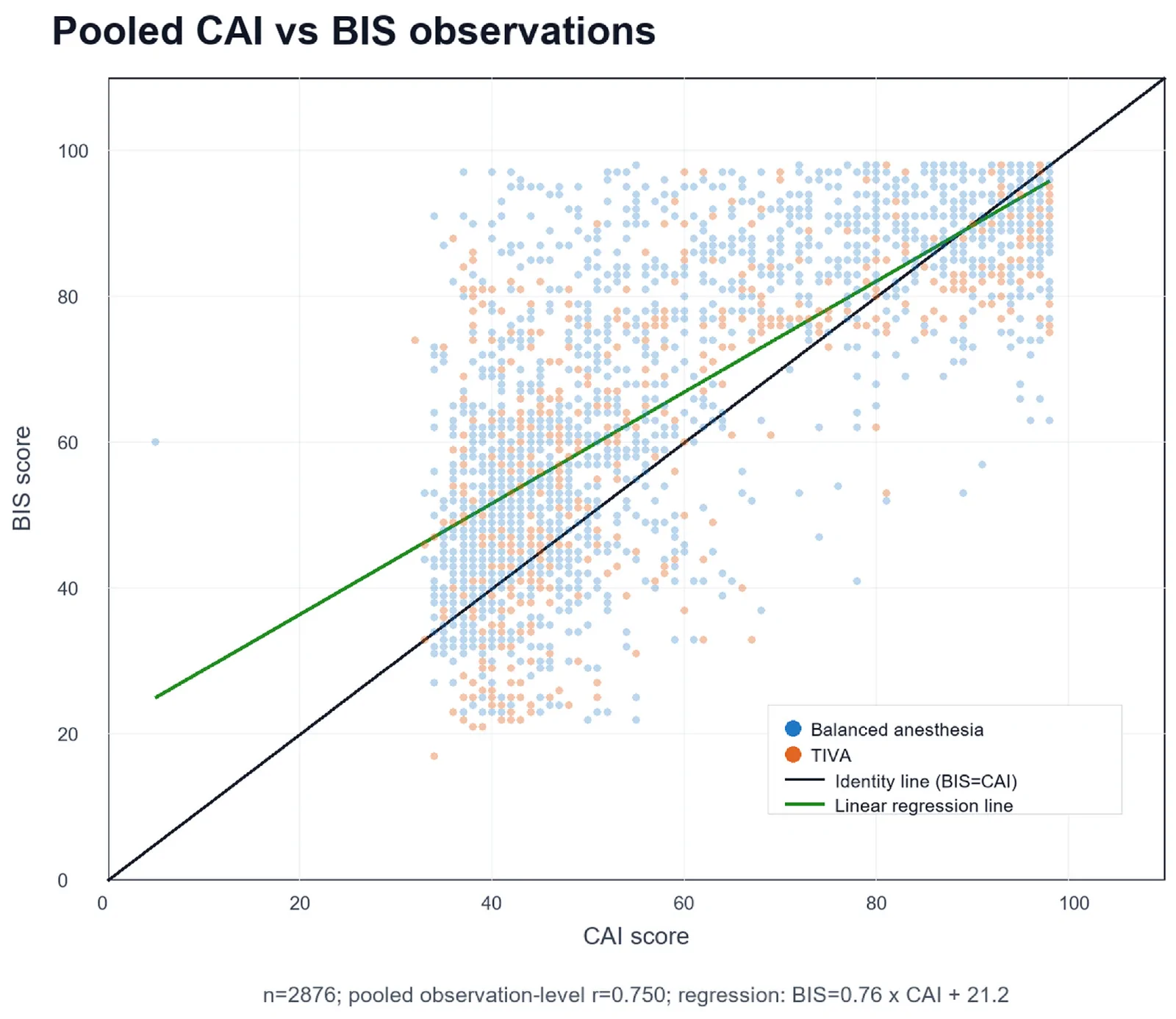
Pooled paired CAI-BIS observations after LOCF handling. Blue points indicate balanced anesthesia, and orange points indicate TIVA. The black line indicates identity (BIS = CAI), and the green line indicates the pooled least-squares linear regression line. The displayed r and regression line are based on pooled observation-level data.

BIS values below 35 were observed in 164 of 2876 BIS observations after LOCF handling (5.7%). At least one BIS value below 35 occurred in 52 of 93 patients (55.9%). Low BIS observations were more frequent in TIVA than in balanced anesthesia (10.0% vs. 4.7%), and at least one low-BIS episode occurred in 12/18 TIVA patients and 40/75 balanced-anesthesia patients. Agreement and divergence summaries are provided in Table 3. The pooled observation-level CAI-BIS relationship is shown in Figure 3.

## 4. Discussion

In this prospective observational study, the prespecified mean patient-level CAI-BIS correlation criterion of r ≥ 0.85 was not met (mean r = 0.778, SD 0.200). The separate pooled observation-level correlation was 0.750. Both indices followed broadly similar perioperative trajectories, but BIS values were generally higher, and marked divergence occurred around intubation. These findings describe concordance and disagreement between two processed EEG outputs; they do not establish the absolute accuracy or superiority of either monitor.

BIS was not treated as a gold standard. It was chosen because it is the most extensively studied processed EEG monitor, has a widely used clinical target range, and guided anesthetic titration in this cohort [1,2,3,4,5,7,8]. Comparative studies have similarly used BIS as a pragmatic reference when evaluating Entropy [9,10], Narcotrend [11], the Cerebral State Index [12], and the Patient State Index [13,14]. Across these comparisons, related indices often tracked anesthetic transitions while retaining device-specific responses or numerical differences, underscoring that association does not establish interchangeability. Thus, BIS provided a clinically relevant benchmark for determining whether CAI moved in parallel with routine BIS-guided care. Because neither index directly measures consciousness and no independent reference standard was included, the comparison permits a relative evaluation but does not provide criterion validation.

The contribution extends beyond the earlier 32-patient comparison by registering 100 participants and analyzing 93 participants across heterogeneous surgical sites, 31 predefined perioperative stages, balanced anesthesia and TIVA, and complementary analyses of patient-level correlation, pooled correlation, agreement, and event-specific divergence. The failure to meet the prespecified r ≥ 0.85 criterion is itself informative and argues against assuming interchangeability. Nevertheless, the study remains a comparative evaluation rather than definitive validation of CAI.

The pooled CAI-BIS correlation during intraoperative maintenance (r = 0.415) was lower than that during induction/intubation or emergence/recovery. This exploratory estimate was based on only three scheduled maintenance checkpoints, and the limited variability of both indices under relatively stable anesthetic conditions may have attenuated the correlation. The low stage-order correlations likewise indicate that neither index exhibited substantial monotonic change across these checkpoints. Nevertheless, genuine algorithm-specific divergence during steady-state anesthesia cannot be excluded. Accordingly, these data do not support CAI-guided anesthetic titration or direct transfer of the conventional BIS target range to CAI without independent prospective validation.

The mean patient-level BIS-CAI difference of 7.6 points shows why directional correlation is insufficient: the BIS range of 40–60 cannot be transferred directly to CAI. The lower correlation observed in the small TIVA subgroup (0.715 vs. 0.794 in balanced anesthesia) is descriptive only and should not be interpreted as evidence of technique-specific CAI performance. In a post hoc analysis, BIS showed a ≥10-point post-intubation increase more often than CAI (73.1% vs. 19.4%). These discrepancies may reflect true physiological responses, drug-specific EEG effects, electromyographic or electrical interference, stimulation, or algorithm-specific processing; because raw EEG, EMG, and signal-quality data were not adjudicated, the present study cannot distinguish among these explanations. In addition, BIS was recorded from the left forehead and CAI from the right, and transient side-to-side differences in frontal processed EEG measurements have been documented [15]. Regional frontal EEG asymmetry may therefore have contributed to the systematic numeric offset or event-specific divergence, and the present design cannot separate this potential acquisition effect from algorithm-specific differences.

Accordingly, CAI should not be presented as superior to or a replacement for BIS. The results support further investigation of CAI as a complementary processed EEG measure, with CAI-specific targets established prospectively. Future studies should compare both indices with independently adjudicated raw EEG features and clinically relevant endpoints such as responsiveness, awareness, recovery, and hemodynamic outcomes.

### Limitations

This study has several limitations. It lacked an independent reference standard for hypnotic state, so comparison with BIS cannot establish the absolute accuracy or superiority of CAI. Anesthetic titration was guided by BIS and usual clinical judgment, which may have favored concordance with BIS-guided decisions. The analysis was limited to recorded index values; raw EEG, EMG, and device signal-quality data were not available for independent adjudication. Consequently, divergence during intubation, emergence, post-extubation, and BIS < 35 episodes cannot be attributed confidently to cortical physiology, EMG activity, electrical interference, or other artifact. Electrocautery, the forced-air warming device, airway manipulation, facial movement, and sensor displacement may have affected recorded values. Before imputation, 8 CAI values and 22 BIS values were missing; LOCF replaced all 8 CAI values and 15 BIS values, while 7 initial BIS values remained missing. Complete-case sensitivity results were nearly identical, but LOCF can still attenuate variability. Opposite-side forehead placement could introduce regional differences. The dataset did not contain scheduled mid-surgery measurements between 5 min after incision and surgery end. Five planned reversal-agent checkpoints and prescreening counts were unavailable. The TIVA subgroup was small and nonrandomized, and the single-center adult cohort limits generalizability.

## 5. Conclusions

The prespecified mean patient-level CAI-BIS correlation criterion of r ≥ 0.85 was not met. BIS served as a pragmatic clinical comparator rather than a gold standard, and the study was not designed to demonstrate CAI superiority. CAI and BIS showed broadly similar perioperative trajectories but differed in absolute values and around intubation. These results support further independent validation of CAI rather than numeric substitution for BIS.

## Figures and Tables

**Table 1:** Baseline and perioperative characteristics.

Variable	All	Balanced	TIVA
*N*	93	75	18
Age, years	51.2 (14.9)	50.3 (15.2)	55.0 (13.2)
Male sex	53 (57.0%)	41 (54.7%)	12 (66.7%)
ASA physical status	2.1 (0.5)	2.1 (0.5)	2.1 (0.5)
BMI, kg/m^2^	25.1 (4.5)	25.4 (4.6)	23.8 (3.9)
Hypertension history	31 (33.3%)	26 (34.7%)	5 (27.8%)
Surgery duration, min	66.6 (49.0)	57.9 (43.5)	102.5 (55.0)
Anesthesia duration, min	104.4 (57.2)	94.7 (52.4)	145.0 (59.9)
Surgery site: abdomen	31 (33.3%)	25 (33.3%)	6 (33.3%)
Surgery site: arm	12 (12.9%)	11 (14.7%)	1 (5.6%)
Surgery site: back	7 (7.5%)	3 (4.0%)	4 (22.2%)
Surgery site: chest	6 (6.5%)	5 (6.7%)	1 (5.6%)
Surgery site: leg	25 (26.9%)	21 (28.0%)	4 (22.2%)
Surgery site: neck	4 (4.3%)	3 (4.0%)	1 (5.6%)
Surgery site: oral	2 (2.2%)	2 (2.7%)	0 (0.0%)
Surgery site: other	6 (6.5%)	5 (6.7%)	1 (5.6%)

Values are mean (SD) or *n* (%). Minor surgery-site categories are retained in the analysis dataset.

**Table 2:** Primary and secondary CAI-BIS correlation analyses.

Analysis	Group	N	Estimate	Median/IQR or 95% CI	r ≥ 0.85
Patient-level r	All	93	0.778 (0.200)	0.845 [0.716, 0.913]	46 (49.5%)
Patient-level r	Balanced	75	0.794 (0.187)	0.854 [0.753, 0.917]	38 (50.7%)
Patient-level r	TIVA	18	0.715 (0.244)	0.828 [0.623, 0.889]	8 (44.4%)
Pooled observation-level r	All	2876	0.750	95% CI 0.734, 0.766	
Pooled observation-level r	Balanced	2318	0.761	95% CI 0.744, 0.778	
Pooled observation-level r	TIVA	558	0.708	95% CI 0.664, 0.747	
Complete-case sensitivity: patient-level r	All	93	0.779 (0.199)	0.843 [0.739, 0.913]	45 (48.4%)
Complete-case sensitivity: pooled r	All	2861	0.751	95% CI 0.735, 0.767	

Patient-level analyses summarize one Pearson coefficient per patient. Pooled analyses use paired stage observations and report Fisher 95% confidence intervals. Complete-case sensitivity analyses exclude all LOCF-imputed observations.

**Table 3:** CAI-BIS agreement and divergence analyses.

Group	N	BIS-CAI Mean Difference	MAE	RMSE	Intubation BIS Spike ≥ 10	Intubation CAI Spike ≥ 10	Any BIS < 35	BIS < 35 Observations
All	93	7.6 (6.5)	12.4 (4.6)	15.3 (5.5)	68/93 (73.1%)	18/93 (19.4%)	52/93 (55.9%)	164/2876 (5.7%)
Balanced	75	8.0 (6.2)	12.2 (4.6)	15.3 (5.5)	55/75 (73.3%)	14/75 (18.7%)	40/75 (53.3%)	108/2318 (4.7%)
TIVA	18	5.7 (7.7)	13.1 (4.6)	15.7 (5.5)	13/18 (72.2%)	4/18 (22.2%)	12/18 (66.7%)	56/558 (10.0%)

MAE, mean absolute error; RMSE, root mean square error. The post hoc intubation-spike definition was an increase of at least 10 index points from intubation to the maximum value recorded 1–5 min after intubation; this was a descriptive, nonvalidated threshold.

## Data Availability

The data supporting the findings of this study are available from the corresponding author upon reasonable request and subject to applicable institutional and ethical requirements.

## Abbreviations

The following abbreviations are used in this manuscript: BIS, bispectral index; CAI, Cortical Activity Index; CRF, case report form; EEG, electroencephalography; EMG, electromyography; IRB, Institutional Review Board; LOCF, last observation carried forward; MAE, mean absolute error; RMSE, root mean square error; TIVA, total intravenous anesthesia.
